# The value of involving patients and public in health services research and evaluation: a qualitative study

**DOI:** 10.1186/s40900-021-00289-8

**Published:** 2021-06-29

**Authors:** Pooja Saini, Shaima M. Hassan, Esmaeil Khedmati Morasae, Mark Goodall, Clarissa Giebel, Saiqa Ahmed, Anna Pearson, Lesley M. Harper, Jane Cloke, Jenny Irvine, Mark Gabbay

**Affiliations:** 1 School of Psychology, Tom Reilly Building, Liverpool John Moores University, Liverpool, L3 3AF UK; 2 University of Liverpool, NIHR ARC NWC, Liverpool, UK; 3 Exeter University Business School, Exeter, UK; 4 ARC NWC Public Advisor, Liverpool, UK; 5 North West Boroughs Healthcare NHS Foundation Trust, Winwick, UK; 6 University of Liverpool, Liverpool, UK; 7 Lancaster University, NIHR CLAHRC NWC, Lancaster, UK

**Keywords:** Public and patient involvement, Capacity building, Participation, Partnership, Collaborative working, Coproduction

## Abstract

**Background:**

Public and Patient Involvement, Engagement and Participation research encompasses working with patients/service users (people with a medical condition receiving health service treatment), public members, caregivers and communities (who use services or care for patients). The Partner Priority Programme (PPP) was developed by the National Health Service [NHS] and National Institute for Health Research Collaboration for Leadership in Applied Health Research and Care [NIHR CLAHRC] NWC to share information and experience on evaluating new services being offered to patients that were seeking to reduce health inequalities, improve people’s health and wellbeing and reduce emergency hospital admissions. This paper seeks to explore an approach developed for involving the public as equal partners within the evaluation and decision-making processes of health and social care services research. The aim of this study was to identify how public advisors were included, the impact of their involvement, and how change occurred within the organisations following their involvement.

**Methods:**

A qualitative approach using focus group discussions was adopted to explore the experiences of two cohorts of participants involved in PPP project teams. Focus groups were held with public advisors (*n* = 9), interns (n = 9; staff or public who received a funded internship for a PPP project), NHS and Local Authority initiative leads (*n* = 10), and academic facilitators (*n* = 14). These were transcribed verbatim and analysed using a thematic approach.

**Results:**

Thirty-two public advisors were recruited to support 25 PPP projects across the Collaboration for Leadership in Applied Health Research and CLAHRC North West Coast [NWC] partner organisations. Three inter-related themes were conceptualised: 1)*“Where it all started - involving public advisors”* identified the varying journeys to recruitment and experiences of becoming a public advisor; 2)*“Steps toward active involvement and engagement”* related to public advisors becoming core team members; and 3) *“Collaborative working to enhance public and patient involvement”* relayed how projects identified the benefits of working jointly with the public advisors, particularly for those who had not experienced this style of working before.

**Conclusions:**

The findings indicate that the PPP model is effective for embedding Public and Patient Involvement [PPI] within health services research, and recommends that PPI is integrated at the earliest opportunity within research projects and service evaluations through the use of support-led and facilitative programmes.

## Plain English Summary

The purpose of involving the public and patients in research is to help them have a say in decisions about healthcare and enable patients or other people with relevant experience to contribute to how research is planned, carried out, and shared with a wider audience. The Partner Priority Programme (PPP) was developed by the National Health Service [NHS] and Local Authority partners to share information and experience on evaluating new services being offered to patients that were seeking to reduce health inequalities, improve people’s health and wellbeing and reduce emergency hospital admissions. In this paper, we explore an approach we developed for involving public advisors (service users/patients, and caregivers) as equal partners within the evaluation and decision-making processes of health and social care services research. The aim of this study was to identify how public advisors were included, the impact of their involvement, and identify the changes organisations made as a result of public advisor involvement. Most projects had not included public advisors in their teams before and initially did not understand how to involve them. By attending scheduled meetings, they had time to learn how to engage with public advisors (and what methods to use to recruit them to be part of their teams). Participants also learned the benefits of including public advisors within their teams. With the help and support provided as part of the programme, public advisors seemed to grow in confidence and take part in teams as equal partners.

## Background

The perils of ignoring the voices of patients, carers and the public were starkly outlined within the Francis Review [1], heralding a need for radical change in health and social care in England. Eleven years on, models and practices for involving these key stakeholders within service redesign, quality improvements, evaluation and research are more evident.

Public and Patient Involvement, Engagement and Participation research encompasses working with patients/service users (people with a medical condition receiving health service treatment), public members, and communities (who use services or care for patients) [2]. Public and Patient Involvement (PPI) can help to tackle health inequalities [3] and enhance immediate links between practice-based evidence and evidence-based methodology through improved clarity of research reports and recommendations [4, 5]. NIHR [6] 10-year plan for public involvement and engagement stated that new staff or new researchers should be *“Going the Extra Mile”* to embed PPI in the culture of NIHR and naturally take on the values and practices of effective public involvement. The six key values and principles proposed in the guidance, which should be used as a supportive framework when embedding PPI, include: *respect, support, transparency, responsiveness, fairness of opportunity*, and *accountability* [6].

Staley and Barron [7] highlighted the importance of reporting involvement which describes the details of what was said and learnt by whom (short term outcomes), what changes were made as a result (medium term outcomes), and the long-term, wider impacts on the research, culture, and agenda. Involvement as *‘conversations that support two-way thinking’* with outcomes for all parties such as gaining new skills, knowledge and values that in turn lead to different choices and changes were important aspects of the evaluation [7, 8]. In a recent study, Giebel and colleagues [9] reported that the extent of PPI and experiences of public advisors resulted in changes in the dissemination of the North West Coast Household Health Survey (HHS). Using methods described by Staley and Barron [7], Giebel et al [9] reported the experiences of public advisors in shaping research dissemination. Public advisors were mostly positive about their involvement in the dissemination of the HHS, but highlighted the need for more transparency and support from researchers [9].

PPI can be seen as ‘tokenistic’ or take place after decisions have already been made, giving limited scope for changes that are informed by equal contributions through PPI. The reported level of PPI differs across research studies with involvement ranging from ‘low’, characterised by researchers asking for views to inform decision-making, to ‘high’, where research is led by service-users [10–12]. Previous research emphasises that public advisors should aim towards being included as part of a partnership rather than a consultant within a research project [10, 13, 14]. A recent systematic review [15] demonstrated a sustained rise in interest and published literature in the evaluation of PPI, including service users and caregivers, particularly its effect on enrolment and retention in clinical trials. However, success from this approach was more evident when all forms of PPI were pooled, such as patients being empowered to be on an advisory committee to full patient partnership in research governance, design, and peer recruitment [16]. Additionally, the effectiveness of PPI was reported to be strongest when people with lived experience of the condition being studied were involved as research partners [12] or PPI was tailored according to the nature of the research to ensure authentic and appropriate involvement [17, 18]. This supports the view of public and patient as experience-based experts who contribute knowledge which complements that of scientists and professionals. Several other benefits have been reported including personal benefits to the public and patients involved [11, 19–21]; capacity building [11, 20]; enhanced quality and appropriateness of research [10, 21–23]; increased involvement from diverse populations in research [18]; development of user-focused research objectives and research questions and information [23, 24]; appropriate recruitment strategies for studies [15]; as well as consumer-focused interpretation of data, and enhanced implementation and dissemination of study results [9, 10, 20, 21], that arise from a greater contribution of PPI.

The National Institute for Health Research (NIHR) Collaboration for Leadership in Applied Health Research and Care North West Coast (CLAHRC NWC) was a collaborative partnership between regional universities, and health and social care organisations (including NHS and local government) which focused on improving patient outcomes through the conduct and application of applied health research. Within the CLAHRC NWC, the PPP was coproduced with NHS and Local Authority partners to establish a new programme focusing on a key shared strategic priority: *“Which out of hospital treatments and care are most (cost) effective in reducing health inequalities, improving population health and wellbeing and reducing emergency admission?”* (CLAHRC NWC Partner Priority Programme 2017–2018) [25]. The PPP was developed in response to the need for evaluation capacity building and for timely, practical, and relevant evidence that could feed directly into current local decision-making. Using evaluation as the vehicle, the PPP also aimed to meet NIHR goals of improving patient outcomes within the region, increasing PPI in research and increasing the research capacity and capability of the health and social care workforce (see Table 1). Finally, by raising awareness of the relevance for service delivery and transformation, the PPP aimed to address the CLAHRC NWC’s goal of reducing the impact of health inequalities.

**Table 1:** Logic Model of NIHR CLAHRC NWC Partners’ Priority Programme (PPP) for Cohorts One and Two

INPUTS	OUTPUTS		OUTCOMES		
	ACTIVITIES	PARTICIPANTS	INTERMEDIATE		ULTIMATE
**What we invest:**	**What we do:**	**Who we reach:**	**What the short-term results are:**	**What the medium-term results are:**	**What the long-term impact(s) is/are:**
Time from partner organisations (NHS, LA, University) and public advisors to attend/support: • Workshop participation (5 sessions) • CIG Support meetings (typically 3 between workshops) • Provision/receipt of any additional training (as required) • Monthly review meetings • Public advisor induction Funding to support public advisors involvement Funding to support Interns Funding for venues and refreshments Website hosting of workshop and training materials.	Methodological input at workshops and CIG Support meetings. Facilitate CIGs. Research training programme for interns (open to all CIGs). Development of bespoke templates and workbook to steer project-level evaluation planning/design. Health inequalities/HIAT awareness raising and support. Public engagement awareness raising and support. Dissemination support and event(s).	NHS and LA Partners • Project evaluation leads (CIG members attending workshops) • Interns • Local partners involved in project-level evaluation teams. Public advisors University partners • Facilitators • Methodological experts	Participating teams would co-learn and be facilitated to co-apply evaluative, evidence synthesis, analytical and reflective concepts and techniques to: • Understand the concept of levels of evaluation and the types of evaluation that are possible and relevant to their project. • Map the context for evaluation surrounding their project including their desired outcomes. • Define the question(s) to be addressed, the approaches and methods relevant to their evaluation. • Design and plan their evaluation in order to inform on-going local developments and change. • Consider how to utilise health equity frameworks within their project. • Understand the role and contribution of public advisors to their evaluation. • Personal learning and development.	• CIGs will have supported project-level Evaluations of initiatives. • Health inequality perspective integrated throughout project-level evaluations of initiatives. • Public advisors fully involved at all stages of the project-level evaluations of initiatives. • Project-level evaluations will have fed into the overall cross-CLAHRC analysis (programme-level) addressing the PPP question as a whole.	By encouraging mixed teams of practitioners, commissioners, patients, public and researchers to work together, and by enhancing their skills, knowledge and expertise, the PPP aims are: • PPP has contributed to evidence informed practice and negotiated change within and across local organisations delivering new models of care. • Capacity developed with our partners to embed evaluation into service transformation and commissioning. • Evaluation seen as a tool for change management – and used as such by partners. • Participants continue to utilise learning in other contexts and to train colleagues in evaluation approaches. • Use of HIAT and consideration of health inequalities is systematized by participants. • Public involvement is systematized in service change and evaluation by participants. • Evaluation as learning - to foster a transparent, inquisitive, and self-critical culture across the CLAHRC NWC Partners. • Knowledge mobilisation infrastructures fostered that are sustainable beyond CLAHRC.
			**INDICATIVE OUTCOME MEASURES – What are we looking for?**		
			• Numbers of participants reached. • A set of clearly defined project evaluation plans from each of the teams • Health inequality is embedded within each element of the Project and evaluation plan. • Public advisors are recruited and included as evaluation team members. • Personal learning and development; whether needs identified prior to workshops have been addressed. • Participants’ experiences/views on the usefulness/pertinence of the support given.	• Evidence and data found, generated, analysed and used to inform the evaluation process at both project and programme level. • Teams supported to implement an evaluation with a focus on tackling health inequalities. • Summary reports providing recommendations to local organisations looking to adopt, develop or adjust new models of health and care. • Public/patients fully involved and engaged in the evaluation process. • A network of peer support is developed. • Plans for dissemination, including: CLAHRC Bites; peer-review journal articles co-produced.	• Overall programme of work completed addressing the objectives of the PPP. • Evidence from following up participants that the learning gained continues to contribute to their work and the work of their organisation/community • Knowledge mobilisation infrastructures are self-sustaining with participants/organisations continuing to work together on change initiatives.

The PPP consisted of a series of evaluation workshops and Collaborative Implementation Groups (CIGs) bringing together initiatives from across the CLAHRC NWC region. The aim of the workshop process was to enable initiatives to carry out robust project-level evaluations that engaged and involved public members as core project team members. Table 2 shows the research projects included within the programme and the number of stakeholders involved within each group. For example, one project included four people from the partner organisation, two academic facilitators and two public advisors. By encouraging mixed teams of practitioners, commissioners, patients, public and researchers to work together, and by enhancing their skills, knowledge and expertise, the purpose of the model was to:

- develop capacity within CLAHRC NWC partners to embed evaluation of service transformation and new models of treatment and care;
- find, generate, analyse and use evidence and data to inform the evaluation process at both project and programme levels;
- support teams to plan and implement an evaluation relevant to the PPP and a focus on tackling health inequalities within the NWC;
- provide a practical and flexible approach to partners’ learning and development requirements;
- develop a system of integrated learning organisations (culture change) linking together similar initiatives across the CLAHRC NWC region.

**Table 2:** Features of projects in Partners’ Priority Programme (PPP) rounds 1 and 2

PPP round	Project title	No. people from partner organization	No. academic facilitators	No. public advisors
**First**	Evaluation of Liverpool GP specification (a quality contract) upon the key areas of healthcare activity, quality of general practice, and patient experience over a 10-year period	4	2	2
	Delivery of high quality primary care at scale and improving access in Blackburn and Darwin	2	1	1
	Evaluation of clinical decision-making in the use of inpatient mental health beds, Cheshire Wirral Partnership NHS Trust	4	1	1
	Identification of factors that contribute to emergency re-admissions to hospital for older patients having received inpatient rehabilitative care (Better Care Now, Blackpool Teaching Hospital)	2	1	2
	Evaluation of the impacts of the Knowsley CVD service, Liverpool Heart and Chest Hospital.	3	1	2
	Evaluation of the impacts of the Knowsley COPD service, Liverpool Heart and Chest Hospital.	3	1	1
	To explore the impacts of a system-wide diabetes care partnership, Liverpool Diabetes Partnership.	3	1	1
	Evaluation of the impacts and effectiveness of each Multidisciplinary Team (MDT), Community Health Services, East Lancashire.	1	1	3
	To map existing service provision and coordination across social care, primary care, and the community and explore accessibility of patient/carer self-management/education, Clatterbridge –Wirral Hospital Trust.	2	1	1
	Evaluation of a community integrated mental health and physical health service, 5 Boroughs NHS Partnership Mental Health Services.	2	1	1
	Evaluation of the impacts of multi-disciplinary integrated Community Care Teams (CCTs), Liverpool Clinical Commissioning Group (LCCG).	2	2	1
**Second**	To explore service users’ experiences of the personality disorders hub and its impact on their overall mental health and wellbeing, Liverpool Mersey Care	3	1	4
	Evaluation of the impacts of the Life Rooms on the recovery of Mersey Care service users, Liverpool Mersey Care.	1	2	1
	To gain an insight into the experience of service users admitted to an inpatient psychiatric ward, Cheshire Wirral Partnership NHS Trust.	6	2	1
	Overarching evaluation framework for public health mental health and wellbeing interventions, Public Health England.	5	2	1
	Evaluation of the Youth Information and Counselling (YIAC) Model, Liverpool CCG).	2	1	3
	Evaluation of the STEP (Succeed, Thrive, Empower Pennine) Service, Blackburn with Darwin CCG and Blackburn with Darwin Council.	5	2	2
	Evaluation of Sefton public sector reform programme; early intervention & prevention (EIP), and community connector project, Sefton Council, Liverpool.	2	2	2
	Evaluating of advice on prescription (social prescribing service): providing income maximisation advice in primary care settings, Liverpool CCG	3	1	2
	Evaluation of a program to enhance wellbeing and quality of Life in Motor Neurone Disease (MND) patients, The Walton Centre, Liverpool	2	2	1
	Evaluation of Wigan Later Life and Memory Service (LLAMS) – improving young onset dementia (YOD) services, North West Boroughs NHS Trust	1	2	2
	Evaluation of Multidisciplinary Team (MDT) working in Integrated Care, Liverpool Heart and Chest Hospital	1	2	3
	Evaluation of the use of home phototherapy as a treatment for physiological jaundice, Liverpool Women’s Hospital	2	2	1
	Evaluation of Telehealth for COPD: Re-design of respiratory services in Liverpool, Liverpool CCG	2	1	2
	Evaluation of a programme for early supported discharge of well, late preterm babies, Lancashire Teaching Hospitals NHS Trust	9	1	2

Within the collaborative implementation groups, participating initiatives are supported by university-based facilitators, with an emphasis on collaborative, co/peer learning by all partners as a group. The PPP model embedded the NIHR principles [6] of *‘Going the Extra Mile’* for providing *respect, support, transparency, responsiveness, fairness of opportunity and accountability*. The concept of communities of practice has also been reflected within the model [26, 27]. Communities of practice are groups of people who share a concern or a passion for something they do and learn how to do it better as they interact regularly [26]. The concept of communities of practice has three dimensions, this includes the community (bring people together through active learning), the domain (shared interest), and the practice (sharing knowledge, methods or tools for learning). Therefore, the PPP reflected these three dimensions in its approach of bringing together practitioners/providers/commissioners, academics, and members of the public creating the community, the shared interest in applied health research, service evaluation creating the domain, and collaborative working through sharing good practice and learning from individual projects creating the practice. This is a process where individuals engaged in thinking together and guided each other through their understanding of a shared problem/aspect, for example, which facilitated the redevelopment of learning rather than literal transfer of knowledge [28]. The aim of this study was to identify how public advisors were included, the impact of their involvement, and how change occurred within the organisations following their involvement.

## Methods

### Participants and sampling

Two cohorts undertook the PPP evaluation workshops and supported the programme, the first during November 2016 to October 2017 and the second from July 2017 to June 2018. All participants involved in a PPP project were invited to take part in the study. Focus groups were held for public advisors (*n* = 9), interns (n = 9; staff or public who received a funded internship for a PPP project), NHS and local authority initiative leads (*n* = 10), and academic facilitators (*n* = 14). All of the focus groups included representation of participants from different projects across the PPP cohorts.

### Design

This study adopted a qualitative approach using focus groups to explore the experiences of both cohorts of PPP. Within the final workshop session, each cohort was invited to participate in scheduled focus group discussions for the particular group they felt was most related to them: focus groups were held for public advisors, interns, NHS and Local Authority initiative leads, and academic facilitators. The reason for conducting separate focus groups was to ensure that participants had anonymity from members of their PPP project group about their feedback and experiences without any concerns about these being relayed to their employer or organisation they were working with. This was intended to facilitate open discussions and focus on specific experiences and roles within individual projects.

### Procedure

The focus groups were scheduled following the final PPP workshops to facilitate participants’ attendance. Based on who attended the final PPP workshop, cohort 1 had four focus groups with interns, public advisor, NHS and local authority initiative leads, and academic facilitators. Cohort 2 had three focus groups with interns, public advisor, and academic facilitators; however, there were no attendees for the scheduled NHS and local authority initiative leads focus group. The study scheduled other focus groups for public advisors and initiative leads at other times following the completion of the PPP programme to enhance further participation, but there was no attendance due to the required travel to locations away from where they lived.

Two focus groups were also undertaken with the PPP design team and research and development managers. As these took place once cohort 2 had begun, the participants reflected on their experiences of the processes within both cohorts of the PPP; thus, holding another set of focus groups with both the PPP design team and research and development managers was not deemed necessary.

Following the principles of the NIHR [6] and Staley and Barron [7], participants were asked to reflect back on their experiences of the PPP, including their starting point, what they learned, how they developed, and their recollection of any “tipping points” [29]. Within the focus group discussions, interviewers asked open-ended questions relating to public advisors involvement in the PPP, including how they were recruited and embedded within project teams and also the impact of their involvement (feeling embedded in the project, what changes resulted from this etc.). Questionnaires were developed following previous work undertaken as part of the Evidence for Change programme [20] and explored the participants’ experiences of the PPP. The interviews were tailored for each of the participant groups; for example, a public advisor may have been asked about their experience of being recruited to a project whereas the project lead may have been asked about their experience of recruiting public advisors to their project. Interviews captured each of the participant’s whole experience of the programme for both cohorts. This allowed further exploration of their participation within the wider CLAHRC NWC programme as well as specifically within the PPP. The progression of each of their roles, participation within the specific project and exit from the programme (if this applied), was also discussed. Interviews were recorded using a digital audio recorder and transcribed verbatim.

### Patient and public involvement

The research question was developed collaboratively with researchers, CLAHRC NWC partners, and public advisors. All public advisors were asked if they wanted to be involved in the evaluation of the PPP and one opted to be involved. The role involved attending meetings to discuss the evaluation, reviewing any circulated documents and providing comments and feedback throughout the process. Some of the reasons why others did not commit to be involved included not having the time, or, being involved in other PPI activities. SA was involved in a series of meetings for this research and the planned analysis. SA is a co-author of this paper and has contributed to the drafting of the paper and the interpretation of the results.

### Training for public and patient involvement (PPI) within the programme

Training for PPI was conducted throughout the workshops and in between when needed by JI, PS, CB or SH. Further support was given by facilitators to individual projects. Previously designed tools (JI and SA) that followed INVOLVE guidelines [2], such as an induction pack for CLAHRC NWC public advisors, were utilised. The induction pack included information on what being a public advisor for CLAHRC NWC may involve, how they could be involved within the wider CLAHRC infrastructure, instructions for how they can claim expenses and payment for their involvement and case studies of other public advisors’ experiences [30].

### Data analysis and interpretation

Findings from the both PPP cohorts were first compared to understand any differences or adaptations between the two cohorts before being synthesised. Transcripts were analysed by RY, SH, JC, KB, PS, MGo and MGa using qualitative thematic analysis [31] to identify themes and sub-themes. The iterative coding process enabled the continual revision of themes based on new information seen in the data until the final classifications of major themes were agreed by the team. The coding frame reflected our a priori interest in the theoretical concepts of transition and resources, and was also developed inductively from the entire data set. The frame helped categorise data in terms of the cultural (e.g. PPI-related values), social (e.g. interpersonal relationships, organisational practices), and psychological (e.g. self-understandings as participants) aspects of PPI (e.g. codes included ‘learning through participation’, ‘trusting professionals’, ‘reflecting upon oneself’). During repeated iterations of coding the team made frequent comparisons across codes and the interview data to develop, review, and refine themes [31] on the basis of the complementarity, convergence, and dissonance of ideas across data sources [32].

### Ethical consideration

Ethical approval was obtained from the University of Liverpool Ethics Research Committee prior to study commencement (Reference number: 2236). All participants were informed about the study via an invitation email that provided details of the study involving focus group discussions, a participant information sheet, and a consent form. All participants provided informed consent and the study was conducted in English.

## Results

Thirty-two public advisors were recruited to support 25 PPP projects (see Table 2). Some public advisors were involved in projects in both cohorts. Table 3 shows the breakdown of participants in each focus group. Table 4 shows the nature of activities that public advisors undertook in PPP1 and PPP2.

**Table 3:** Participants who took part within each focus group

Participants	Cohort 1 (Nov 2016 to Oct 2017) n (%)	Cohort 2 (Jul 2017 to Jun 2018) n (%)
**Public advisors**	5/16 (31)	4/27 (15)
**Interns**	5/11 (45)	4/14 (29)
**Partner leads**	6/11 (55)	0/14 (0)
**Facilitators**	8/8 (100)	8/12 (67)
**Research & development managers**	NA^a^	4/4 (100)
**PPP design team members**	NA^a^	6/6 (100)

^a^NOTE: Partner leads, research & development staff and the PPP design team members were interviewed about both cohort 1 and 2

**Table 4:** Nature of activities that public advisors undertook in PPP 1 and 2

Activity Type	Number of activities	Number of public advisors
**Governance**	43	10
**Training**	58	16
**Research design**	68	26
**Undertaking research**	43	16
**Evaluation**	24	7
**Dissemination**	15	7
**Recording podcasts**	8	17

Following the thematic analysis process, three inter-related themes were conceptualised as reflecting the corpus of this material. The themes illustrated how public advisors were introduced, integrated and involved in the PPP projects. The first theme *“Where it all started – involving public advisors”* discussed the varying journeys to recruitment and experiences of public advisors becoming involved in the programme. The second theme related to public advisors becoming a core team member and was conceptualised as *“Steps toward active involvement and engagement”*. The third theme was *“Collaborative working to enhance public and patient involvement”* and related to how projects identified the benefits of working jointly with the public advisors for a shared goal and purpose; particularly for those who had not experienced this style of working before. Each of these themes is developed below.

### “Where it all started” – involving public advisors

Participants discussed the avenues and processes by which a public advisor was recruited for the individual projects. For example, some involved: a formal recruitment process (advertisement of a role description followed by a formal application and an interview); others described direct recruitment of members of the public already actively involved in NHS or charity organisations; recruitment via recommendations from other professionals or project teams; or via the CLAHRC NWC public advisor register:

“*So they went through an open, firm, transparent process for people to apply for it and you had to put an expression of interest forward*” (PPP1- FGD6-Public Advisor)

Interns and project leads reported that the idea of having a public advisor as a research team member and the process in which it happened was a new concept. Participants from cohort 1 reported being unclear about the role of a public advisor. Having to recruit a public advisor without understanding the role made the process of recruitment challenging for some:

“*My most frustrating part has been the public advisor recruitment and that was a very long and drawn out process. Firstly, we didn’t realise it was like mandated to have a public advisor because our evaluation had already been set before we started PPP … but it felt like it was doing something tokenistic to tick a box for PPP as opposed to how they would then come into an evaluation that was already underway*”. (PPP1- FGD3-Project Lead)

Some participants suggested that it would have been easier to recruit a public advisor from the CLAHRC NWC register than having to find their own public advisor. Other participants reflected on projects from cohort 1 that struggled to recruit a public advisor from within their local settings, and following failed attempts sought a CLAHRC NWC registered public advisor:

“*We were asked to find our own public advisor and then after a number of months once we had some conversations, CLARHC actually arranged [public advisor recruited from CLAHRC NWC] for us and took away that headache, as that process, it wasn’t something we were familiar with*” (PPP1-FGD3-Project Lead)

This was a learning point for cohort 2 which was addressed by ensuring the process for public advisor recruitment was initiated early on. For example, for cohort 2, the gap between the first workshop (where teams were asked to engage with public advisors) and the second workshop was two months, enabling each project team additional time to recruit. Furthermore, CLAHRC NWC facilitators supported project teams with recruitment of public advisors by developing a sample role description. Table 4 describes the range of activities public advisors were involved in and how they could be utilised within each of the individual projects. This facilitated useful discussions and most importantly the sharing of lived experiences from cohort 1 as these public advisors then presented as part of the cohort 2 PPI training:

“*I think we were aware of some of the complexities of getting a public advisor and so we could use that experience to go ‘actually yeah this is what the other team did, why don’t you try that’ and so I think we also had an experience, which we could then utilise as a facilitator*” (PPP2- FGD2-Facilitator)

However, figuring out the role of the public advisor within health services research was a reoccurring issue reported by participants. This was a challenge for both public advisors and other participants; at times some participants found it difficult to make a distinction between involving a member of the public as a research participant and engaging a public advisor as a member of the project team:

“*A lot of the things that are evaluated are services that people are going to potentially be recipients of. So, for this, those individuals, that are usually public advisors, wouldn’t be the recipients of this work. It would actually be people that would use the evaluation tool not necessarily the people who use the interventions. So I think that was really difficult.*” (PPP1-FGD1- Intern)

Facilitators in this study reported that in cohort 2, project teams were encouraged to involve the public advisor in defining aspects of their own role where specific skills may be shared. For example, some public advisors had experience and skills of completing statistical analysis with large data sets from their career that they wished to utilise within this voluntary public advisory role. Even though this facilitated discussions, some public advisors reported that this could still cause uncertainty because there were no clear expectations set by the project team who were unsure about how the public advisor may be involved within the project. For example, data governance and what non-NHS people are ‘allowed’ to do with the data. In other studies, project teams would utilise these experiences and skills and the public advisor would be involved in many aspects of the research such as data collection through conducting semi-structured interviews. Although the process of defining the role and recruitment of the public advisor to each project team could be challenging, as each team progressed with regular PPP training workshops and project team meetings, the PPI was improved and enhanced. Participants mentioned that over time the role of each public advisor became clear.

To address the process of defining the role and recruitment of public advisors, one public advisor believed that an experienced PPP public advisor would be in a better position to deliver an induction to new public advisors and support them through the process if they were to join the team at a later stage:

“*They just give you all these forms and things, ‘you’ve got to fill in this, you’ve got to do this, you’ve got to do that, you’ve got to say this’, I said that didn’t work for me. Now after say 8-10 weeks I offered myself, I said ‘look I really don’t think you’re going to be able to explain this to the public advisor because you’re not in that position, you can’t do that’ because they didn’t understand exactly what was going on eight weeks ago either. So I said ‘look I should be doing the training for your next public advisor not you, because I’ve been through it’*”. (PPP2- FGD1- Public Advisor)

The PPP encouraged each project to recruit more than one public advisor to their team in line with INVOLVE guidance [2], and over half achieved this. Most participants felt that having more than one public advisor was not only beneficial to the project but for the public advisor too, especially those who were new to the role. This enabled them to feel confident to ask questions from their peers to enhance their understanding and supported them to become more involved:

“*Luckily I got a very nice person working with (public advisor name) and I was like, kept asking a thousand questions, what to do, what is that, what is that so they helped me a lot*” (PPP2-FGD1 –Public Advisor)

“*If I had gone with the previous public advisor and they had explained it to me I would have understood much better than from the professional*” (PPP1-FGD2-Public Advisor)

Although, project teams were encouraged to involve public advisors from the outset, there seemed to be a lack of clarity of what this meant, particularly for those who were new to this concept. Over the two cohorts, learning was shared about the processes that did and did not work and improvements were made to provide more facilitation, training and clarity on the role of a public advisor. The next theme developed this further and highlighted the methods used to increase the involvement and engagement of public advisors through the support of the programme and individual facilitators.

### Steps toward active involvement and engagement

The PPP encouraged and supported projects to involve public advisors as equal peers within the research team. This included actively engaging and involving each public advisor throughout the different stages of the project (see Table 4), i.e. project design, data collection (conducting interviews, data analysis and research dissemination), recording a podcast, presenting at conferences, co-writing journal articles, and public facing summaries of the research known as ‘CLAHRC BITEs [Brokering Innovation Through Evidence]’ (https://clahrcprojects.co.uk/resources/bites). Participants reported on the process that resulted in them becoming actively engaged (core members) within a project. Project leads and research and development managers stated that when they joined the PPP their lack of understanding about the public advisor role and lack of clear defined evaluation objectives (for their individual projects) hindered their ability to adequately engage with their public advisor:

“*I think in the beginning the person doing the project didn’t really know what they were doing and it was hard to invite someone else along because you didn’t really know what they’ll be doing*” (PPP1-FGD4- Research & Development Manager)

However, within cohort 2, some of these feelings were less evident, particularly for those who were involved in the both cohorts and who built on their experience:

“*I think we had a better way of expressing pubic involvement, which fitted a little bit better with their world having been through the experience before*” (PPP2-FGD2-Faciltors)

“*The majority of time it’s been people [partners] coming to me to ask me if I would be part of them and that’s because you have been identified as a public advisor and that just helped you to kind of get more opportunities*” (PPP2-FGD1-Public Advisor)

The PPI evolved over the two cohorts following feedback and led to changes within the PPP; development of role descriptions for public advisors (LH, RY, PS and JI) in cohort two, public advisors from cohort one acting as mentors for new public advisors in cohort two, and simplification of the payment processes for public advisors are examples of such evolution. For new teams within cohort 2, the addition of role descriptions and presentations from public advisors involved in cohort 1 helped somewhat but more support was needed for some projects from facilitators in navigating the PPI recruitment process:

“*With some people you know involving public advisors was like something that was really brand new to them. They weren’t sure how to engage them in terms of research and we were able to tell them how to do that”.* (PPP2-FGD2- Facilitators)

Some public advisors reported that even though they felt they had the skills to equip them to engage in different parts of the projects (such as writing, reviewing reports, or interview skills), they withheld because they were not clear on what was expected of them at the initial stages. Many felt that they needed better direction to facilitate their engagement:

“*My involvement was fairly light, that isn’t because I didn’t want to get involved. I remember the first occasion I met the team at the hospital and I said what are the boundaries, I don’t know what the public advisor does apart maybe from sitting in the corner and smiling and nodding, but I feel as if I could achieve more than that, what are the boundaries?*” (PPP1-FGDD6- Public Advisor)

“*My intern, she was actually brilliant, she was the one who was actually spoon feeding me, which at that time I did really need that spoon feeding approach. I wasn’t sure what CLAHRC was all about, I was given loads of information and that was too much overload for me … having sat down with the intern she … said well this is what we need, this is what we’re doing and it was a step-by-step approach. It was spot on for me but that’s the way I learn anyway*”. (PPP1-FGD6- Public Advisor)

Having public advisors involved as part of the project team and having support from the facilitators, helped in enhancing understanding and capacity for PPI. Many participants discussed how they recognised and valued the public advisor contribution to the project. Public advisors continued engagement and involvement within PPP workshops, CIGs, project meetings and other project activities. This enabled them to become more confident in their contribution as a public advisor:

“*Because I know a bit more now and I feel a bit more confident to say yes I have got a proper valuable role to play in this, it’s not just lip service*”. (PPP1-FGD6- Public Advisor)

Participants highlighted different activities public advisors contributed towards including project design, assessing health inequalities through the use of the health inequalities assessment tool [33], data collection (conducting interviews, distributing surveys), data analysis (reviewing transcripts, coding data), and being involved in dissemination activities (poster and oral presentations, reviewing papers for publication). Through their involvement, public advisors developed research capacity that enabled them to become a core lead, for example, with project data collection and analysis.

“*The public advisor really took to NVivo (qualitative data analysis software package) and the partner didn’t get it so much and it’s the public advisor who anonymised all the transcripts. I don’t think they expected the public advisor to say I will do that. It’s moving people out from just seeing a patient as a generalised statistic of a person to individuals who have their own skills and experiences*.” (PPP1-FGD2 – Facilitator)

Involvement within the PPP created further opportunities for public advisors as they were able to get involved in other CLAHRC NWC projects and in subsequent PPP cohorts. Many project leads and facilitators recognised that some public advisors could build their capacity to be involved or lead future research projects and aided the process through encouraging and facilitating them to continue their development. Their involvement was not limited to being a public advisor as two progressed to becoming interns, leading a project in the following round of the PPP and all took up different roles across the CLAHRC NWC such as being part of the wider public advisory forum:

*“My journey hasn’t ended. I’m in a very fortunate and unique position where I have progressed from public advisor into the internship and involvement. I am fortunate in that way and I’ve been very supported both from the project side and from the CLAHRC university side” (PPP2- FGD1- Public Advisor)*

*“I’ve become a health ambassador so you probably see posters here and there, so it has opened up a lot of opportunities outside of CLAHRC for me” (PPP2-FGD1- Public Advisor)*

The progression of public advisors being integrated into the wider infrastructure of the CLAHRC NWC was evident. The growth and development of public advisors along with the other participants was illustrated within this theme. The beneficial gains of collaborative working are highlighted further in the next theme.

#### Collaborative working to enhance public and patient involvement

The PPP workshops and CIGs brought together different project members as peers with a common area of interest. Participants found that their involvement was beneficial as it created the opportunity to explore the perspective of others and reflect on their own, especially, for PPI:

“*At first it just seemed well why are we doing this, are we just doing this because we’ve been told to and its part of CLAHRC but actually I think they were all you know really surprised at how much value the patients brought to their evaluation in terms of the (Project01)”* (PPP1-FGD2-Facilitator)

The opportunity of being involved in the PPP created time and space away from their usual environment, be that a workplace or home, that enabled partners to have a deeper reflection on PPI and working on developing their approach and moving away from tokenistic involvement. Within the PPP model the importance of having PPI was highlighted and introduced at the first workshop through: specific PPI training; sharing of knowledge on how other projects had included PPI successfully; facilitating the recruitment of public advisors which could be a novel experience for some projects; and, the facilitation of the public advisors involvement within a project. However, it was vital to ensure that the public voice was equal to the practitioners/commissioners/academics in meetings. The importance and value of the public advisor contribution within projects was highlighted:

“*I wouldn’t say tokenism but to a certain extent they were doing it, but it was a slightly difference focus and it wasn’t really involving them [public advisor name]. It was very much more stakeholder meeting, ‘what do you all think’. Whereas, actually I think one of the positives from this [PPP] is we have actually got some of the groups to start thinking, if you have a public member as part of your team the difference it makes is very positive.*” (PPP1- FGD2-Facilitator)

“*We bring a different perspective because everybody’s brain thinks differently but we need to ensure public advisors are brought on to add value and not to compete with the academics in some cases*” (PPP2-FGD2-Public Advisor)

Most participants reported that being part of the PPP was a journey of learning that enabled them to reflect and understand PPI more deeply. The CLAHRC public advisory forum led by the CLAHRC public engagement lead (JI) provided another aspect of support for the public advisors recruited to the PPP projects and to all stakeholders within the PPI training elements of the programme. Participants managed to see the value public advisors brought to the team which enabled them to contextualise their understanding of PPI more:

“*I’ve learnt that actually, although I was converted I maybe sometimes thought well what is the value of somebody coming to this meeting and doing this, I understand now that sometimes it’s just about asking the question and thinking about things a bit differently*” (PPP1-FGD4-research & development manager)

This section highlights how each participant's experience was influenced by the context and depended on the working of groups within individual projects. The capacity building element, via the PPP model for involving key stakeholders within service redesign, evaluation and research, was established over the two cohorts. Equal partnership was achieved across some of the projects, with some public advisors going on to lead new projects within CLAHRC NWC or becoming involved as equal partners within other health-related groups across the region. Although, it is worth noting that not all projects progressed at the same rate and some needed more facilitation and guidance than others. Such projects, may have been offered more meetings and help from the CLAHRC facilitators and PPI team. The approach was to encourage CIGs to recruit PPI members from their partner organisations and networks rather than relying on recruiting them from the cadre already involved as public advisors in the CLAHRC who would instead offer guidance and mentorship.

## Discussion

This study provides an insight into the PPP model and the way of working (via CIGs and extensive support in evaluation and methods) in building PPI capacity amongst NHS and Local Authority partners, academics, and members of the public. All participants in this study found the PPP model effective in enhancing their understanding of the value of PPI in applied research and supporting the development of innovative methods of actively involving members of the public in all aspects of a research project. The study identified key facilitators that influenced the level in which public members were integrated in evaluation. This included: enhancing the individual and organisational understanding of PPI; defining the role of public members in research and incorporating that into a transparent recruitment and induction process; developing the project team’s capacity in utilising public advisors’ skills; and supporting public members to becoming core team members. The key values and principles proposed within the ‘Going the Extra Mile’ report [6] were embedded throughout the PPP as there was clear respect for public advisors, support provided through training and induction, transparency and responsiveness from project leads involving them in all aspects of the work thus creating fairness of opportunity and evidence of accountability within reports provided to the PPP.

The structure in which the PPP was delivered was important in building PPI capacity amongst its members, particularly as PPI can sometimes be complex and understanding the process of PPI in theory can be challenging for some. Participants reported that the learning with the public members and others, and exploring and trialing of how best to use PPI, supported them transferring learning from theory to practice. The learning from cohort 1 brought about valuable lived experiences that participants found useful in enhancing the learning and practice of PPI in cohort 2; for example, earlier recruitment of public advisors, co-designing the public advisors’ role, and reflecting on how to better involve public advisors. This process of ‘thinking together’ was important in cultivating PPI in the PPP (as communities of practice), which facilitated the sharing of tacit knowledge (such as PPI) through individual reflection [27].

**Fig. 1:**
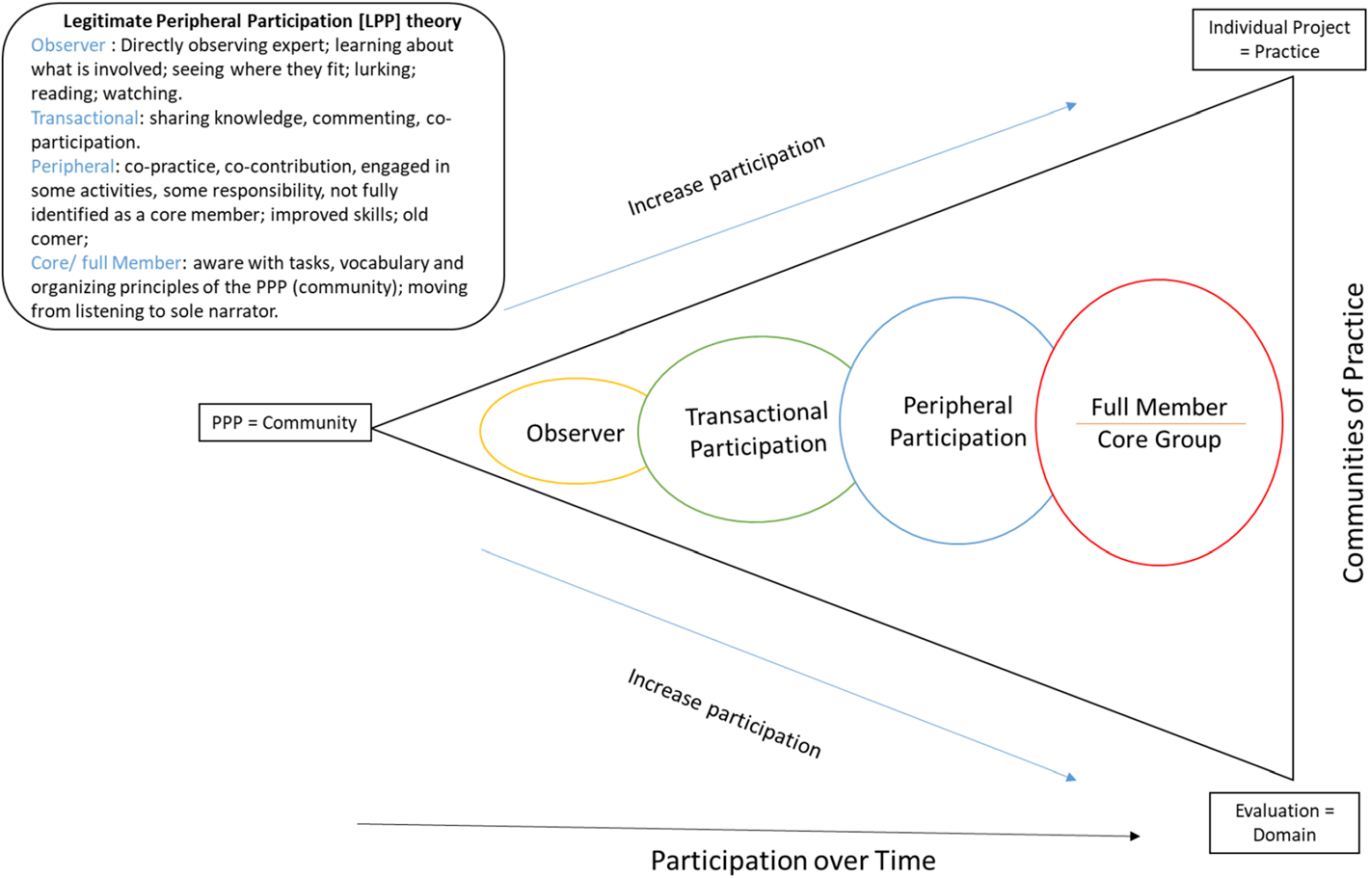
PPI within the Partner Priority Programme (PPP) through the lens of reflecting the Communities of Practice and Legitimate Peripheral Participation theory [26]

Importantly, the three dimensions of communities of practice [26, 27] highlighted earlier, with the community being the PPP, domain being the evaluation, and the practice being the individual project, was the platform in which public advisors were facilitated to become actively involved as core members (Fig. 1). For example, some reported needing to be observed as newcomers before actively participating in the team project. Thus, when reflecting on the legitimate peripheral participation theory [26] that describes the process of participation within a community, public advisors were moving from being an observer to participating as a core member as participation increased over time (Fig. 1). The legitimate peripheral participation theory [26] is embedded within the PPP communities of practice dimensions (Fig. 1), whereby the level of involvement experienced by participants and exploring the learning that occurs as one participates in communities of practice [28]. The learning process of newcomers is a path from being an outsider to becoming a core member, which includes undertaking more complex activities, using more advanced practices, taking over functions that are more relevant and central for the community, and adopting roles requiring a deeper understanding that are more constructive for the goals of the community [34]. In this study, public advisors progressed from observers to active (transactional and peripheral) participants in undertaking tasks such as co-designing project outputs, collecting and analysing data, reporting and dissemination of findings, and, for some, leading on research projects as interns. However, how rapid this process happened varied for public advisors. Some reasons for the variation may be due to a public advisor joining the project late in its development, a lack of clarity in their role or place within the project and resulting in the requirement for additional support to move them from being “the observer” to “active participation”. Participants reported that early recruitment of public advisors supported quicker learning for project teams on the process and value of embedding PPI. Similar to previous studies, over the course of the project, the project leads, interns and facilitators found that their own understanding of patient-oriented research deepened [10].

This study provides a clear illustration that exemplifies the formation of a partnership between public advisors and professionals/academics within a project team. Key facilitators and barriers were identified, such as training and support on how to recruit and involve public advisors for partner organisations, for the full integration of public advisors in applied health research. As previously suggested by Curwen and colleagues [11], the PPP provides guidance on simplifying procedures, such as for payments, and establishing a moving-on support system to help people access regular employment and gain full social inclusion. This study has developed a pathway to undertake the NIHR’s [6] vision for increasing the prominence of PPI in research and the numbers taking part, particularly in the North West Coast of England. Within the CLAHRC NWC partners, some researchers and clinicians now automatically include public groups when they start developing their research protocols and they have more knowledge in how to recruit people who may want to be involved as public advisors. This is now a pre-requisite for research teams in the CLAHRC successor organisation the NIHR Applied Research Collaboration NWC, and is embedded in systems such as the equity toolkit used in all projects (www.hiat.org.uk) [30].

Some PPI has been found to be less effective in research projects [15]. Across the PPP projects, there was a greater impact on those public advisors who had more support from project teams. As stated in previous research [12, 16, 17] and due to the structure of the PPP, all participants could be supported and trained in how to involve their public advisor but the outcome of their joint working was specific to each project. However, the facilitation could have enhanced the high level of public advisor involvement across the projects. Similar to Gray-Burrows and colleagues [17], this study has indicated the importance of understanding the needs of public advisors when developing models to involve people from different population groups in their projects.

### Strengths and limitations

This study has a number of strengths. Firstly, the PPP enabled the development of new innovative ways of involving a range of public advisors within numerous CLARHC NWC partner projects simultaneously. From our knowledge, this is the first study to date to embed public advisor within up to 13 partner projects (cohort 1) and then 12 projects (cohort 2) concurrently. Similar findings have been reported within the Evidence for Change programme that embedded public advisors into four projects simultaneously [20]. Secondly, the model provides an innovate solution to support the full integration of public members within health and social care design and evaluation and within health services research. Public advisors were instrumental in the translation of scientific concepts into accessible ideas for the non-scientist through writing lay summaries and presenting project findings within both community settings and conferences. Similar findings have been reported previously [7–9, 20, 21]. Thirdly, this study demonstrates ways to integrate full public engagement in evaluations through a structured programme of workshops and meetings, over a set period of time, with clear project expectations and deadlines. Over the two cohorts, the PPP created clear goals and definitions for projects embedding public advisors within their evaluation as partners. The importance of partnership working has been emphasised in other studies [10, 13, 14, 20, 21]. Fourthly, there was diversity within each team of stakeholders who had different interests and different decision-making outcomes. PPI included multiple perspectives rather than demographic characteristics or sampling frame. Projects were encouraged to include PPI on the different health services/areas. Thus, working collaboratively with diverse involvement of members in each team and increasing inclusivity in the decision-making processes such as the design, delivery and use of the services being evaluated (Table 4). Public advisors from different health areas were involved in all of the projects (Table 2), providing an opportunity for more varied PPI within the wider partner organisations and the CLAHRC NWC public advisory forum. However, more information on participant demographics should be collected for future research. Lastly, within the programme there were clear core features, activities, and mechanisms about how to involve public advisors and how their roles may evolve within the individual projects and the wider CLAHRC NWC infrastructure. Similar to previous findings [10], participants were reflective about the unspoken values and power imbalances underpinning patient and public involvement. This study emphasised the mutual impact for both public advisors and other stakeholders on personal and organisational levels and highlighted that public advisors were invested on to ensure that the evaluation research for individual projects answered questions that mattered to them and their communities. Within the PPP, public advisors became a well-resourced component of the evaluation and not just as a tokenistic aspect of the work as shown in Table 4. Eleven years on since the recommendations of the Francis Review [1], this study provides a model for effectively involving the public as equal peers/partners in health and social care evaluation, reform and redesign.

However, some limitations remain. Firstly, we cannot rule out the possibility that focus groups were not representative of all public advisors who were recruited within the PPP, in terms of background, ethnicity, gender, caring duties, and of patients/former patients. There was a low recruitment rate for public advisors and following some reflection for future studies we would complete one-to-one interviews with public advisors rather than focus groups to try to improve their participation. We did not ask participants why they opted not to participate, as this would be against the ethical considerations for the study. However, we think that the low rates may be due to the location of the focus groups being held at a distance difficult for some public advisors to travel to. Secondly, facilitators were included as participants within the focus groups which may have caused bias and less objectivity. Thirdly, the work has been created by undertaking only one small study involving two iterations of the PPP within the region. We therefore recommend that the model created is further validated by applying it to other studies conducted elsewhere within the UK.

### Meaning of the study: possible implications for adoption

There was evidence of a positive effect of the PPP model with an improvement in embedding PPI. Firstly, this study highlighted the personal and organisational benefits to involve public members as equal partners within individual projects. This was supported by the positive reflections from all participants and could indicate that the model may have been effective in providing a structured and supportive environment where this type of learning and activity could take place.

One of the requirements of being included as part of the PPP was that each project had to involve PPI. This gave an incentive to involve public advisors and indirectly gained value and benefits to the organisations involved. However, as mentioned above, the extent to which public advisors contributed was largely dependent on their commitment, contribution, and how they were integrated within each team. Future projects should therefore consider time for team members to understand the skills of their public advisors to ensure that tasks should be specifically tailored to public advisors’ needs and expertise. Furthermore, training should be an integral for project teams, as it may help to empower them to take control of incorporating PPI.

The evidence for recent PPI in the UK has tended to rely on single studies [15]; thus providing limited evidence of impact for health services research/evaluation. This model appears to have been effective and sustained long term, as it improved within round two. These findings indicate that the PPP model is effective for embedding PPI within health services research/evaluation, and recommends that PPI is integrated at the earliest opportunity within research projects and service evaluations through the use of support-led and facilitative programmes.

## Conclusion

The findings indicate that the PPP model is effective for embedding PPI within health services research, and recommends that PPI is integrated at the earliest opportunity within research projects and service evaluations through the use of support-led and facilitative programmes.

## Data Availability

The datasets generated and/or analysed during the current study are not publicly available due to it being qualitative interview accounts of their experience in being involved in Partner Priority Programme Projects, but are available from the corresponding author on reasonable request. All of the transcripts have been anonymised and no names or places will be available within the data.

## Abbreviations

CLAHRC: Collaboration for Leadership in Applied Health Research and Care
FGD: Focus group discussion
HHS: Household health survey
NHS: National Health Service
NIHR: National Institute for Health Research
NWC: North West Coast
PPI: Public and Patient Involvement
PPP: Partner Priority Programme
LA: Local Authorities
HIAT: Health Inequalities Assessment Tool
